# Durable Effect of Radioactive Iodine in a Patient with Metastatic Follicular Thyroid Carcinoma

**DOI:** 10.1155/2012/231912

**Published:** 2012-09-23

**Authors:** Aubrey A. Carhill, Rena Vassilopoulou-Sellin

**Affiliations:** ^1^Division Endocrinology, Department of Medicine, Baylor College of Medicine, One Baylor Plaza Mail Stop 185, Houston, TX 77030, USA; ^2^Department of Endocrine Neoplasia and Hormonal Disorders, Unit 1461, The University of Texas MD Anderson Cancer Center, 1400 Pressler, Houston, TX 77030, USA

## Abstract

*Objective*. Thyroid cancer is the most common endocrine malignancy and fastest increasing of all cancers in both men and women in the United States. Traditionally, differentiated thyroid cancer (DTC) carries a good prognosis when diagnosed early, but increasingly patients are presenting with late-stage disease and bone metastasis which carries a poor prognosis. Treatment of DTC involves surgical resection followed by radioactive iodine (RAI), which conventionally is thought to reach maximal effectiveness between 6 and 12 months following treatment. We report a case and review the literature surrounding long-term effect of radioactive iodine treatment in metastatic thyroid carcinoma. *Methods*. Patient clinical encounter and the literature review. *Results*. We describe a 49-year-old woman with symptomatic metastatic follicular thyroid cancer (FTC) to the spine and radiographic evidence of spinal cord compression who was effectively treated with RAI. Her initial serum thyroglobulin (Tg) levels following total thyroidectomy were 1,343 ng/mL which dramatically dropped to less than 100 ng/mL following RAI. Forty-three months following treatment with RAI, she has experienced complete resolution of her symptoms and continues to maintain persistently low-thyroglobulin levels of less than 100 ng/mL. *Conclusions*. RAI is believed to reach peak efficacy within 6–12 months; however, little has been reported regarding the long-term duration of benefit. This case demonstrates that the benefits of RAI therapy may be enduring, even in patients with widely metastatic thyroid cancer. It suggests in clinically stable patients with declining thyroglobulin after treatment, that there may not be an immediate need for additional therapy as RAI treatment may provide lasting effects.

## 1. Introduction

An estimated 56,000 new cases of thyroid cancer will be diagnosed in the United States in 2012 [1]. Although the incidence of tumors <1.0 cm has risen with improved detection techniques, the incidence of tumors of any size, especially tumors >4 cm with distant metastases, has also risen [2]. Increasingly, patients are presenting with late-stage disease, particularly aggressive-differentiated thyroid cancer (DTC) with bone metastases [3, 4].

DTC is the most common form of thyroid cancer and includes papillary thyroid cancer (PTC; >85%) and follicular thyroid cancer (FTC; 5–10%). Most patients with DTC undergo surgical resection followed by treatment with radioactive iodine (RAI) and thyroid hormone suppression [5]. Although RAI is believed to reach maximum effect at 6–12 months, relatively little is known regarding its long-term duration of benefit. We present a patient with metastatic FTC in whom RAI has continued to show clinical benefit after 43 months. 

## 2. Case Report

A 49-year-old woman presented to The University of Texas MD Anderson Cancer Center for evaluation of metastatic thyroid cancer. Seventeen years earlier, she had undergone left thyroid lobectomy for a reportedly benign 4 × 4 cm colloid nodule. She had been in good health since then, regularly participating in marathons; approximately 1 year prior she noted progressive left hip pain that began to interfere with her training. The pain remained unresolved after physical therapy and she began to develop new symptoms of numbness and tingling in her left lower extremity. 

**Figure 1:**
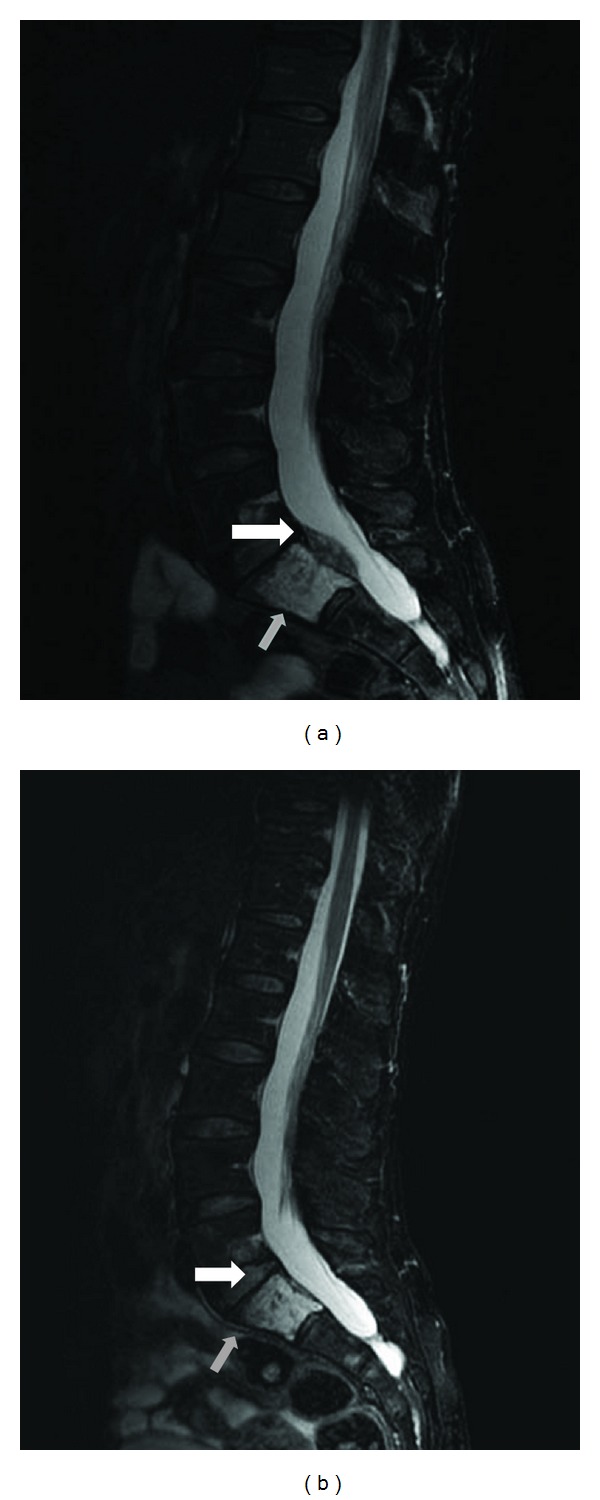
Magnetic resonance imaging (T2 sagittal image) performed at the patient's initial clinical presentation shows S1 metastasis (gray arrow) and cord thecal sac compression (white arrow) (a). Magnetic resonance imaging (T2 sagittal image) performed 3 years after the patient's second RAI treatment shows complete resolution of the tumor (b).

Magnetic resonance imaging of the patient's spine and pelvis revealed a 6.3 × 4.1 mm lytic sacral lesion in S-1 compressing the spinal canal (Figure 1(a)) and an iliac tumor highly suggestive of metastatic disease. She underwent computed tomography of the chest, abdomen, and pelvis, which revealed multiple subcentimeter pulmonary nodules and low-attenuation lesions in the liver. Subsequent biopsy of the sacral lesion revealed metastatic FTC; immunohistochemical staining of the specimen was positive for thyroid transcription factor-1.

The patient reported only mild anterior rib and left hip pain with numbness radiating down her left leg. On physical examination, she had an absent left thyroid lobe, no palpable masses in the right thyroid lobe and no lymphadenopathy. Neurologic exam was grossly normal with full power and intact sensation throughout, normal reflexes, and no clonus. She ambulated without difficulty and only had some mild pain on palpation of the sciatic notch. 

Neurosurgical evaluation noted significant epidural disease which had transferred pressure to the left S1 nerve root, causing mild radicular symptoms. However, because the sacral lesion was not causing significant bone destruction or evidence of spinal instability, it was felt there was no immediate indication for surgical intervention. Neurosurgical recommendations included treating the patient with selective external beam radiotherapy (EBRT) or RAI. Head and neck surgery also evaluated the patient for completion thyroidectomy to facilitate treatment of distant metastatic disease with RAI. 

Presurgical laboratory findings included a serum thyroglobulin (Tg) level of 2,632 ng/mL, a thyroid-stimulating hormone (TSH) level of 0.15 uIU/mL, and no interfering Tg autoantibodies. She underwent right lobectomy and isthmusectomy with left paratracheal/thyroid bed exploration and right paratracheal excisional lymph node dissection. Pathology revealed a microscopic 2-mm focus of PTC confined to the right thyroid lobe and no evidence of lymph node involvement. Postsurgically, the patient's serum Tg level dropped to 1,343 ng/mL with a suppressed TSH of 0.17 mIU/mL. 

**Figure 2:**
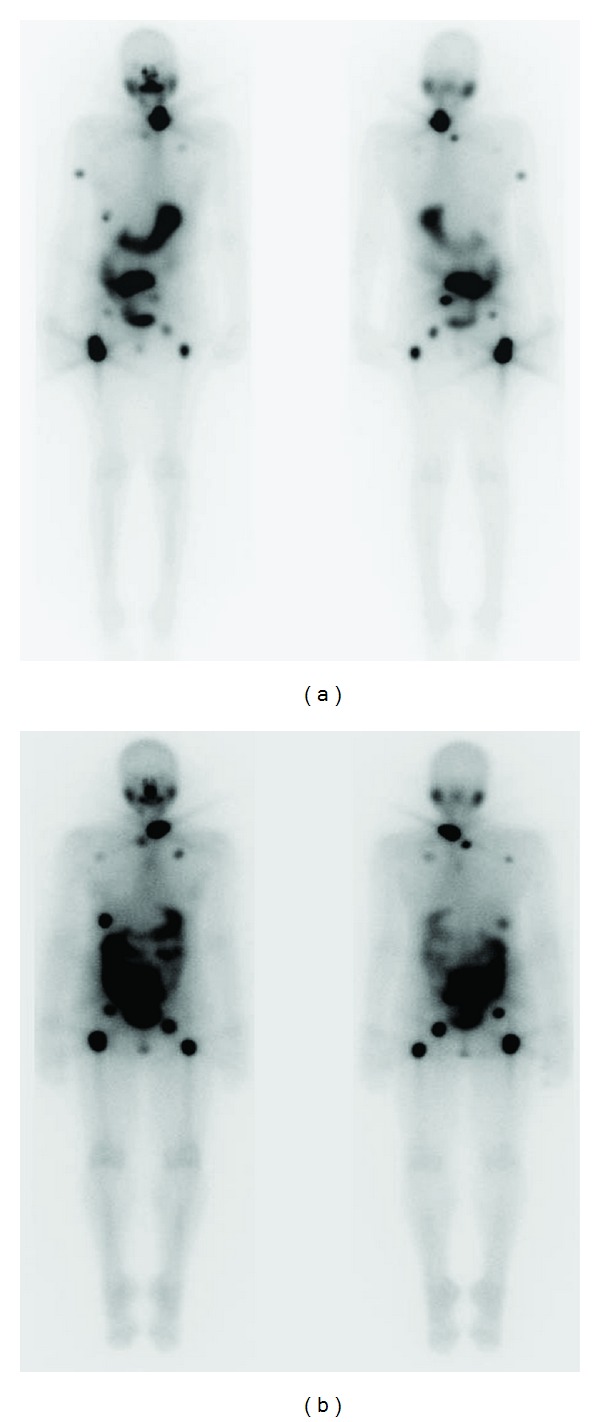
Whole-body radioactive iodine (RAI) scan after the first (a) and second (b) RAI treatments.

Adjuvant therapy included recombinant human TSH (rhTSH) preparation followed by RAI therapy with 203 mCi of I-131. Her posttreatment I-131 scan revealed foci of uptake in the left thyroid bed, lungs, humerus, ribs, acetabulum, and femurs (Figure 2(a)). The patient also received EBRT to the sacral tumor. 

Six months after completion thyroidectomy, her serum Tg level was 205 ng/mL, which increased to 885 ng/mL with rhTSH stimulation. Diagnostic RAI whole body scan revealed persistent multifocal iodine-avid lesions in the thyroid bed, thorax, abdomen, and pelvis. Repeat therapy was administered with 200 mCi of I-131. Post treatment I-131 scan showed uptake in the same areas (Figure 2(b)). 

**Figure 3:**
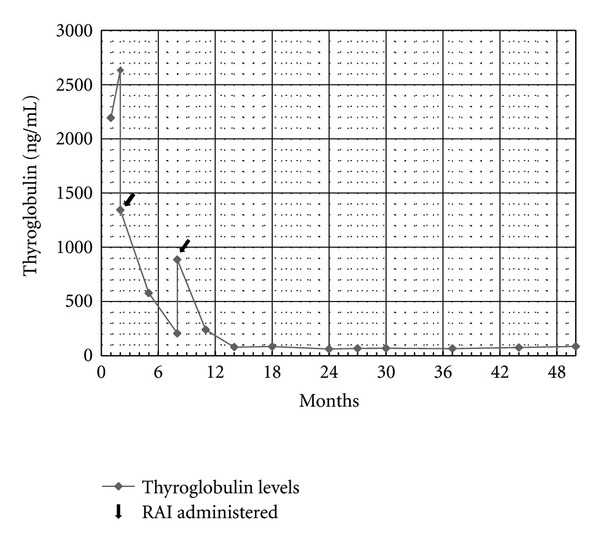
Trend in serum thyroglobulin level over time.

Since completion of her second I-131 treatment, the patient has been treated with thyroid hormone suppression therapy only. Forty-three months after initial RAI treatment, she remains active and asymptomatic with complete resolution of her spinal compression (Figure 1(b)). Her serum Tg level has continued to decline and remains consistently low (<100 ng/mL; Figure 3); recent diagnostic imaging has not revealed evidence of disease progression. Of note, although the patient's Tg dropped dramatically, her levels were still detectable; we reserved further RAI until further evidence of progression was noted, especially since it was the patient's strong wish to limit radiation exposure.

## 3. Discussion

In general, DTC is associated with good prognosis and near-normal life expectancy [6]. The overall 10-year survival rate of patients with well-differentiated thyroid cancer is between 80% and 95%, but overall survival rate of patients who develop distant metastases is 40% [6–9]. Patients with bone metastases have an even lower 10-year overall survival rate, ranging from 0% to 34% [7, 8, 10], with mean survival duration of about 4 years [11]. FTC accounts for less than 15% of all DTCs; however, the incidence of bone metastases in FTC patients ranges from 7% to 20% [12], which is markedly higher than that in PTC patients (1–7%) [13]. Factors contributing to bone metastases may include FTCs ability to spread hematogenously, to synthesize molecules that enhance attachment to bone matrix, and to secrete angiogenic compounds that promote bone reabsorption, such as receptor activator of nuclear factor-*κβ* ligand [14]. 

The patient's presenting symptom was bone pain caused by metastatic FTC. Based on our findings, we believe that her cancer was likely present at the time of her initial lobectomy 17 years prior, though we were unable to review original pathology specimen. The initial lobectomy was likely effective surgical treatment, but because FTC has a tendency to spread hematogenously, it is possible at the time of her initial surgery she had extrathyroidal extension of microscopic disease. This is consistent with the results of her completion thyroidectomy, which contained a small foci of micropapillary carcinoma and no discernible FTC in the neck. 

Because no prospective randomized controlled clinical trials of RAI for the treatment of bone metastases in DTC patients exist, data regarding the efficacy of RAI in such patients come from retrospective studies. In these studies, nearly all DTC patients with skeletal metastases received RAI. The largest retrospective study [7] included 2,200 DTC patients, 394 of whom had lung and/or bone metastasis; of those 394 patients, 201 had metastatic FTC. The patients underwent total thyroidectomy and received 100 mCi of I-131 adjuvantly. After initial treatment, patients in whom I-131 scans revealed I-131 uptake in the lungs and/or bones, patients on T4 suppression who had detectable serum Tg levels, and patients on T4 suppression with serum Tg levels >5 ng/mL during withdrawal received an additional 100 mCi of I-131. Patients with bone metastases also received 30 Gy of EBRT to the affected regions. The patients in whom I-131 scans revealed I-131 uptake in the lungs and/or bones had better prognosis than the patients in whom I-131 scans did not reveal such uptake, and the risk of death was highest in patients with multiple bone metastases, who had a 10-year overall survival rate of 14%. 

A different retrospective review of 107 DTC patients with skeletal metastases treated with RAI showed that the rate of partial and complete remissions in patients younger than 45 years (62.5%) was higher than in those older than 45 years (49.0%) [15]. I-131 elicited complete remissions in 75% of younger patients with 3 or fewer bone lesions, suggesting that I-131 could be used with curative intent in certain patients. A long-term outcome study of 444 DTC patients with distant metastases who received RAI showed similar results [13]. 

The prominence of skeletal involvement in morbidity from FTC has been well described. Large studies have shown that RAI reduces recurrence [16, 17] and mortality in patients with DTC and can be used to effectively treat distant metastases [7, 18]. RAI, while not always curative, has also been found to relieve pain and markedly reduce serum Tg levels [19]. In patients with metastatic FTC, RAI constitutes primary therapy for small-volume regional residual disease and isotope-concentrating distant metastases.

The emergence of angiogenesis-inhibiting therapies has expanded the number of treatment options available to patients with metastatic DTC. For many practitioners, the focus of treatment of metastatic disease has shifted from conventional therapy to newer therapies, resulting in shorter time intervals between the use of RAI and subsequent use of angiogenesis inhibitors. However, it is important to note that antiangiogenic therapies traditionally elicit poor responses in bone metastases; rather, the majority of confirmed responses to antiangiogenic therapy in patients with DTC in clinical trials per Response Evaluation Criteria in Solid Tumors have occurred in soft tissue metastases [20]. RAI elicits responses in both bone and soft tissue metastases. 

RAI is generally thought to reach peak efficacy within 6–12 months; however, relatively little has been reported regarding the long-term duration of benefit or exact mechanism of action on a cellular level for late-onset effects induced by radiation. But, as this case demonstrates, the benefits of RAI therapy may in fact be very durable, even in patients with widely metastatic thyroid cancer. This case suggests in clinically stable patients in whom serum Tg levels decline following RAI treatment, additional therapy to be deferred as RAI may continue to provide lasting benefit.
